# Involvement and readiness of fellows from Papua New Guinea’s Field Epidemiology Training Programme in the COVID-19 response, 2020–2021

**DOI:** 10.5365/wpsar.2023.14.2.989

**Published:** 2023-06-24

**Authors:** James A Flint, Joanne Taylor, Tambri Housen, Barry Ropa, Bernnie Smaghi, Laura Macfarlane-Berry, Celeste Marsh, Alois Pukienei, Mathias Bauri, David N Durrheim

**Affiliations:** a Hunter New England Health, New Lambton, New South Wales, Australia.; b University of Newcastle, Newcastle, New South Wales, Australia.; c National Department of Health, Port Moresby, Papua New Guinea.; d World Health Organization Representative Office for Papua New Guinea, Port Moresby, Papua New Guinea.; e Department of Health, Autonomous Bougainville Government, Autonomous Region of Bougainville, Papua New Guinea.; f Western Highlands Provincial Health Authority, Western Highlands Province, Papua New Guinea.

## Abstract

**Problem:**

Fellows of the Papua New Guinea Field Epidemiology Training Programme (FETP) were part of the national coronavirus disease (COVID-19) response. However, the specific activities and challenges experienced by fellows in the field were unknown.

**Context:**

The advanced FETP cohort commenced just before the COVID-19 pandemic and all fellows were involved in the response. The advanced fellows participating in this review represented a cross-section of the country’s public health workforce.

**Action:**

A review was conducted to better understand the scope of activities undertaken by FETP fellows, identify the challenges experienced and assess how well the programme prepared fellows for their COVID-19 response roles. A facilitated discussion based on the World Health Organization COVID-19 intra-action review methodology and an online survey was conducted with advanced FETP fellows.

**Outcome:**

The fellows made important contributions to the national COVID-19 response by assuming leadership positions at all levels of government, leading training activities and applying core field epidemiology competencies in surveillance and response activities. The programme had prepared them well for the response, giving them the confidence and skills to undertake a diverse range of response roles.

**Discussion:**

The FETP review of the COVID-19 response in Papua New Guinea highlighted the role and influence of the fellows during the pandemic response. Fellows were able to apply core field epidemiology competencies across a range of roles. The recommendations derived from this review will be instructive for the FETP specifically and the COVID-19 response generally.

## PROBLEM

Graduates and fellows of the Field Epidemiology Training Programme of Papua New Guinea (FETPNG) were part of the national coronavirus disease (COVID-19) response. However, the specific activities and challenges experienced by FETP fellows in the field were not known. Given the important role of field epidemiologists in emergency response, the FETP faculty conducted a review to understand what worked well, what worked less well, the scope of activities undertaken by fellows during the COVID-19 response, how prepared fellows felt, their confidence in performing key field epidemiology tasks during the response and what FETPNG could do better to prepare fellows for future infectious disease emergencies.

## CONTEXT

The COVID-19 pandemic has tested public health emergency response capacity across the world. The first case of COVID-19 was confirmed in Papua New Guinea (PNG) on 6 March 2020 and the country has experienced multiple waves since that time, relying heavily on international and domestic border control measures as well as contact tracing, quarantine and isolation to suppress transmission and preserve health systems. (*1*, *2*) As of 22 August 2022, 44 861 confirmed cases of COVID-19, including  664 deaths, were reported in PNG. (*3*)

FETPs are supervised, on-the-job, competency-based training programmes for public health professionals. They train field epidemiologists to collect, analyse and interpret public health information, using evidence to take action and save lives. The skills of locally trained field epidemiologists are well suited to support public health emergency response activities. (*4*) As health security concerns have grown globally, FETPs have become increasingly recognized in global, regional and national preparedness and response mechanisms. (*5*) Field epidemiologists are identified as important human resource requirements for implementation of the International Health Regulations (2005), or IHR (2005). (*6*, *7*) The Global Health Security Agenda, launched in 2014 to support IHR (2005) implementation, highlights workforce training as a key element in strengthening health security. (*8*) FETPs are a key part of training this health security workforce. Regionally, the Asia Pacific Strategy for Emerging Diseases (APSED III) has identified the importance of FETPs in progressing IHR (2005). (*9*)

PNG has been running an intermediate level (9-month) FETP since 2013 (*10*) and recently initiated an extended 18-month programme, known as the advanced FETPNG (aFETPNG). As of July 2022, there were 94 intermediate FETP graduates working across all 22 provinces of the country and 17 fellows enrolled in aFETPNG.

The aFETPNG cohort commenced in 2019 just before the COVID-19 pandemic, and work in 13 of PNG’s 22 provinces (59%). They represent all levels of the government’s public health workforce, with fellows recruited from district (*n* = 7), provincial (*n* = 9) and national levels (*n* = 1). The substantive roles of fellows included surveillance officers, health extension officers, district health managers, disease programme managers, provincial disease control officers, the FETP convenor and a provincial deputy director of public health.

## ACTION

### Facilitated discussion

A 1-day review was held with aFETPNG fellows during their second face-to-face training workshop. We adapted the World Health Organization (WHO) COVID-19 intra-action review methodology, (*11*) framing discussions with FETP fellows around WHO’s emergency response pillars which were used to guide a country’s COVID-19 response. (*12*) The pillars we focused on were:

Risk communications and community engagement (pillar 2);
Surveillance, case investigation, laboratory (pillars 3 and 5);
Case management and infection prevention and control (pillars 6 and 7); and
Operational support and logistics (pillar 8).

Facilitated discussions identifying what went well and what went less well during the COVID-19 response were held, which included a root cause analysis. (*11*, *13*) Findings from the root cause analysis were used to develop recommendations for action.

### Online survey

Understanding the contribution of aFETPNG fellows during the COVID-19 response, their role, how well prepared they felt and their confidence in performing key field epidemiology tasks during the response was carried out through an online survey. (*13*) The survey also asked how FETPNG could better prepare fellows for future infectious disease emergency responses.

## OUTCOME

### Facilitated discussion

The findings from the facilitated discussion and key recommendations derived from root cause analysis were organized into four groups based on the WHO pillars (**Table 1**).

**Table 1:** Summary of what worked well, what worked less well and key recommendations for the advanced  Field Epidemiology Training Programme of Papua New Guinea, based on root cause analysis, April 2022

Risk communications and community engagement		
Worked well	Worked less well	Recommendations
Using established systems and community structures Partnerships with key stakeholders Community leaders trained and engaged in COVID-19 awareness Risk communications training for health-care workers (HCWs) at provincial and district levels Good political influence in the community Other partners helped develop information, education and communication (IEC) materials that were easy to understand by the community	Misinformation about COVID-19 vaccination and the impact this has on COVID-19 vaccination and routine immunization HCWs spreading false rumours about the virus and COVID-19 vaccination Lack of established partnerships with communities affected communication and engagement efforts Provincial communication officers not always available Limited use of local languages in IEC materials	Establish and maintain strong working relationships with community leaders and partners Establish high-quality training-of-trainers strategies to ensure HCWs at all levels are knowledgeable across response needs Establish recruitment strategy at provincial level to ensure adequate professional health staff to raise public health awareness alongside risk communication experts Continue to work with and build relationships with partners
**Surveillance, case investigation, laboratory**		
**Worked well**	**Worked less well**	**Recommendations**
Roll out of rapid antigen test kits Provincial-level management support for surveillance activities Opportunities afforded to Field Epidemiology Training Programme (FETP) fellows to apply surveillance skills Purchase of two-way radios for surveillance teams Training of health extension officers at district level to collect specimens Capitalizing on COVID-19 surveillance to strengthen other reporting systems Proactive response supported by appropriate legislation	Turnaround time for polymerase chain reaction (PCR) results (2–4 weeks) Turnaround time for whole genome sequencing Lack of training in data management No dedicated data management officers at provincial or district levels for COVID-19	Roll out COVID-19 rapid antigen tests at all facilities, including aid posts Ensure supply of rapid antigen tests is adequate Develop a sensitization programme to highlight the value of surveillance to management within the province
**Case management and infection prevention and control**		
**Worked well**	**Worked less well**	**Recommendations**
When available, rapid antigen tests helped with timely case detection/diagnosis Improved health facilities (e.g. construction of new wards and isolation facilities, instalment of incinerators, etc.) Creation and dissemination of treatment protocols Engagement of mental health counsellors	Limited or no patient transport available No expertise to deal with mental health problems Standard treatment protocols not always available, confusion around the use of ivermectin Insufficient human resources for case management and infection prevention and control Poor coordination and cooperation between clinical and public health response Poor compliance with case isolation	Direct funding and resources to boost health-care workforce Provide staff incentives for additional responsibilities Target educational resources to promote vaccination among HCWs Build new isolation facilities or separate COVID-19 wards with dedicated staff to work in them Ensure resources are allocated to home isolation monitoring Strengthen and invest in sustainability of call centres in all provinces (for example, integrate the call centre with the disaster office) Offer staff incentive packages and infection prevention and control training for those who work with COVID-19 patients
**Response, operational support and logistics**		
**Worked well**	**Worked less well**	**Recommendations**
Integration of COVID-19 response with other programmes Establishment of rapid response teams (RRTs) to support the response Strengthened emergency operations centres at the provincial level Coordination of funding available for COVID-19 response Involvement of partners/commercial properties to support response needs	Staff shortage – inadequate staffing resulted in multitasking, exhaustion and mental stress Waste management issues (e.g. non-functional incinerators) Delay in receiving funds for the response Disruption to routine services, including routine childhood immunization Funding impacts on other programmes Poor compliance with control measures (mask wearing, physical distancing, isolation, quarantine, vaccination)	Establish and allocate funding for a RRT in every province; use existing workforce to formulate RRTs Ensure there is a provincial budget for COVID-19 response and outbreaks with programme-based budgeting Establish processes at provincial level to facilitate rapid mobilization of financial and human resources in response to public health emergencies (with minimal impact on routine services) Provide targeted education and incentives to promote vaccination of HCWs at all levels

### Online survey

Fifteen (88%) aFETPNG fellows responded to the survey. All 15 (100%) were involved in the COVID-19 response in PNG. When asked about their involvement in COVID-19 throughout 2021, just over half (53%; *n* = 8) reported working full time on the response. Of those not in a full-time role, 13% (*n* = 2) worked on the response 3–4 days per week and 33% (*n* = 5) 1–2 days per week.

The most common COVID-19 response roles undertaken included leading surveillance activities, providing advice to stakeholders, leading rapid response teams (RRTs), contact tracing and conducting training. The majority (80%; *n* = 12) of fellows received specific training to support them in their COVID-19 response roles. Almost all fellows (93%; *n* = 14) were involved in training others in support of the COVID-19 response, with fellows conducting an average of four training activities  (range 1–15) in 2021. The 14 fellows collectively trained over 700 individuals.

Core FETP competencies, such as disease surveillance, outbreak response and data analysis, were all highlighted as being useful in preparing fellows for the COVID-19 response. Fellows also identified that the FETP provided them with confidence, enabling them to fill leadership roles, conduct public speaking and influence decision-makers.

“Decision makers have confidence in me presenting analysed data on COVID-19.”

“As an FETP fellow, I have been appointed incident manager – I took a lead in surveillance, contact tracing, risk communication and community engagement.”

“There is respect for the [FETP] course.”

“There is recognition of FETP grads who are identified to take lead roles in the response.”

“From the FETP training – we could actively participate as a team lead in RRT, conduct contact tracing, case investigation and surveillance – across all areas of response.”

Fellows felt most confident supporting or leading case investigation and contact tracing activities, and least confident supporting or leading risk communication, community engagement, specimen handling and shipping, and infection prevention and control activities (**Supplementary Table 1**).

Areas for strengthening the response capacity of graduates included further training on tools to support surveillance, data management, analysis and interpretation, risk communications and community engagement, psychological first aid, management and leadership during public health emergencies, and the establishment of RRTs. Fellows highlighted a need for more careful consideration and inclusion of gender issues when responding to emergencies and commented on connectivity challenges associated with virtual training.

Most fellows (93%; *n* = 14) reported that the intermediate and advanced FETPs were very helpful in preparing them for the COVID-19 response, while one respondent (7%) indicated the programmes were moderately helpful. Half (*n* = 7) of the fellows indicated that their manager was very aware of their skills as a field epidemiologist, 36% (*n* = 5) of managers were somewhat aware and 14% (*n* = 2) were not aware. Most of the fellows (79%; *n* = 11) indicated that their skills in field epidemiology were well used by their managers during the COVID-19 response.

When asked what could be done to improve the use of FETP graduates and fellows by management, the following themes emerged: (i) the need for management to recognize the potential of field epidemiologists and make use of them in leadership positions; (ii) the creation of designated field epidemiology positions within the public service; (iii) FETP sensitization training for managers; and (iv) the need for FETP fellows and graduates to appropriately manage up, including proactively presenting their surveillance and project findings to management.

## Discussion

The COVID-19 review highlighted the role and influence of aFETPNG fellows during the pandemic response. Fellows were able to apply core field epidemiology competencies across a range of roles. The diversity of their roles highlights the value and versatility of field epidemiologists in public health emergencies. While the majority of fellows found the FETP training very helpful in preparing them for a pandemic response, they identified areas for improvement.

Based on the findings from the facilitated discussion and the online survey, the faculty prioritized the following actions:

revise the intermediate and advanced FETPNG curricula to include additional training on areas highlighted by fellows, especially risk communication and community engagement;
develop supplementary training, tools and resources to enable fellows and graduates to master core FETP competencies; fellows identified eLearning modules (provided in both offline and online formats), further refresher training opportunities, and a written technical manual with PNG examples;
develop mechanisms to support graduates in the ongoing application of FETP knowledge and skills in the workplace through activities such as individual and group-based projects for graduates and ongoing mentorship (including during outbreak response activities);
develop and deliver a sensitization training programme for senior management to promote the best utilization of field epidemiology graduates in the workplace; and
advocate for the creation of designated field epidemiology positions within the public health service, providing a clear career pathway for graduates.

This FETP COVID-19 review was limited to fellows enrolled in the advanced FETP and did not include feedback from fellows or graduates of the intermediate FETP. Thus, these findings are not representative of all FETP fellows and graduates and cannot be generalized to the whole FETPNG population.

This COVID-19 review supports a culture of ongoing reflection and evaluation. The recommendations are instructive for FETPNG specifically and the COVID-19 response generally. Findings from this review support previous work focusing on workforce issues during emergency responses. (*4*, *14*, *15*) This review has highlighted the important contribution of the FETP fellows during the COVID-19 response, and the need for the programme to adapt to better prepare PNG’s field epidemiology workforce for future challenges.
